# Depression-related weight change and incident diabetes in a community sample

**DOI:** 10.1038/s41598-021-92963-w

**Published:** 2021-06-30

**Authors:** Eva Graham, Tristan Watson, Sonya S. Deschênes, Kristian B. Filion, Mélanie Henderson, Sam Harper, Laura C. Rosella, Norbert Schmitz

**Affiliations:** 1 Department of Epidemiology, Biostatistics, and Occupational Health, Faculty of Medicine and Health Sciences, McGill University, Montreal, QC Canada; 2 Douglas Mental Health University Institute, 6875 LaSalle Boulevard, QC H4H 1R3 Verdun, Canada; 3 ICES, Toronto, ON Canada; 4 School of Psychology, University College Dublin, Dublin, Ireland; 5 Jewish General Hospital, Lady Davis Institute, Montreal, QC Canada; 6 Department of Medicine, McGill University, Montreal, QC Canada; 7 Department of Pediatrics, Université de Montréal, Montreal, QC Canada; 8 Centre de Recherche CHU Sainte-Justine, Montreal, QC Canada; 9 School of Public Health, Department of Social and Preventive Medicine, Université de Montréal, Montreal, QC Canada; 10 Epidemiology Division, Dalla Lana School of Public Health, University of Toronto, Toronto, ON Canada; 11 Department of Psychiatry, Faculty of Medicine, McGill University, Montreal, QC Canada; 12 Department of Population-Based Medicine, Institute of Health Sciences, University Hospital Tuebingen, Tuebingen, Germany

**Keywords:** Diseases, Risk factors

## Abstract

This cohort study aimed to compare the incidence of type 2 diabetes in adults with depression-related weight gain, depression-related weight loss, depression with no weight change, and no depression. The study sample included 59,315 community-dwelling adults in Ontario, Canada. Depression-related weight change in the past 12 months was measured using the Composite International Diagnostic Interview—Short Form. Participants were followed for up to 20 years using administrative health data. Cox proportional hazards models compared the incidence of type 2 diabetes in adults with depression-related weight change and in adults with no depression. Adults with depression-related weight gain had an increased risk of type 2 diabetes compared to adults no depression (HR 1.70, 95% CI 1.32–2.20), adults with depression-related weight loss (HR 1.62, 95% CI 1.09–2.42), and adults with depression with no weight change (HR 1.39, 95% CI 1.03–1.86). Adults with depression with no weight change also had an increased risk of type 2 diabetes compared to those with no depression (HR 1.23, 95% CI 1.04–1.45). Associations were stronger among women and persisted after adjusting for attained overweight and obesity. Identifying symptoms of weight change in depression may aid in identifying adults at higher risk of type 2 diabetes and in developing tailored prevention strategies.

## Introduction

Major depressive disorder, or depression, affects approximately 264 million people worldwide^1^. Depression is also associated with an increased risk of chronic conditions, including type 2 diabetes, as well as poorer functioning, disability, and early mortality^1,2^. Adults with depression have an 18% increased risk of developing type 2 diabetes compared to adults without depression^3^. However, major depressive disorder is a highly heterogeneous condition and can include symptoms that have opposing associations with metabolic health, such as depression-related weight gain and weight loss. In a sample of community-dwelling adults with recent depressive symptoms, 12% of men and 17% of women with depression experienced symptoms of weight gain while 11% of men and 17% of women experienced symptoms of weight loss^4^. It is not clear whether adults with depression with distinct symptoms of weight change, including weight gain and weight loss, differ in their risk of type 2 diabetes.

Cross-sectional evidence among adults with depression and symptoms of appetite change, closely related to symptoms of weight change, suggests that depression with increased appetite is associated with poorer metabolic functioning. Adults with depressive episodes that included increased appetite had higher BMI values, a higher number of metabolic syndrome components, and increased markers of inflammation^5,6^. Adults with depressive symptoms that included decreased appetite had lower BMI values, smaller waist circumferences, fewer metabolic syndrome components, and lower levels of inflammation^5^. These findings suggest that adults with depression and increased appetite, and those with depression-related weight gain, may have poorer metabolic health and may be more likely to develop type 2 diabetes.

Evidence from longitudinal studies has focused on clusters of depressive symptoms rather than individual symptoms. In the DSM-V, depressive episodes with atypical features include symptoms of mood reactivity and at least two of the following: increased appetite or weight gain, hypersomnia, leaden paralysis, and interpersonal rejection sensitivity^7^. Melancholic features of depression include either loss of energy or lack of mood reactivity as well as three of the following: decrease in appetite or weight loss, depression that is worse in the morning, early morning awakening, psychomotor change, and excessive guilt^7^. In a community population, adults with a history of depressive episodes with atypical features had higher increases in fasting glucose over 5 years compared to adults with no depressive episodes^8^. Adults with melancholic features of depression did not have higher increases in fasting glucose over this period^8^. Atypical depression is also longitudinally associated with other indicators of metabolic dysregulation, including increased obesity, metabolic syndrome, and inflammatory markers^9,10^. Findings from these studies suggest that adults with depression-related weight gain, a component of atypical depression, may have a higher risk of type 2 diabetes. Notably, atypical depression is more common in women and associations between depressive symptoms and diabetes may be stronger among women^11,12^. However, prior work has not examined sex differences in associations between depressive subtypes and metabolic outcomes.

It is also important to consider that depression-related weight gain may be associated with type 2 diabetes due to the development of overweight and obesity^13^. Some evidence suggests that weight gain is associated with an increased risk of type 2 diabetes largely as a result of attained weight, overweight and obesity^14,15^. However, other studies indicate that long-term weight gain is associated with the onset of type 2 diabetes independent of attained weight^16,17^. Further evidence is needed to clarify the role of depression-related weight change and the incidence type 2 diabetes when accounting for attained overweight and obesity.

The primary objective of this study was to compare the incidence of type 2 diabetes over 20 years in community-dwelling adults with recent depressive episodes that included symptoms of weight gain, weight loss, or no weight change, and in adults without recent depressive episodes. Our primary analysis also estimated sex-stratified associations. We expected that adults with depression-related weight gain would have an increased incidence of type 2 diabetes compared to all other groups. As a secondary objective, we estimated these associations when accounting for attained overweight and obesity after a depressive episode.

## Participants and methods

### Study population

This study included participants from two health surveys in Canada, the National Population Health Survey (NPHS; 1996) and the Canadian Community Health Survey (CCHS), cycles 1.1 (2000–2001) and 2.1 (2003). These surveys collected cross-sectional health information from separate representative samples of Canadians living in private dwellings, with further details described elsewhere^18^. This study pooled data from all three survey waves and included participants aged 18 and older living in the province of Ontario, Canada^19^. NPHS and CCHS participants were linked to administrative follow-up data for up to 20 years that provided information on diabetes incidence, death, and contact with the healthcare system. Informed consent was obtained from all participants for participation in these surveys and for linkage with administrative data. Ethics approval for this project was granted by the Research Ethics Board of the Douglas Mental Health University Institute. All methods were performed in accordance with the relevant guidelines and regulations and with the Declaration of Helsinki.

A total of 74,159 adults responded to the NPHS or CCHS surveys, were successfully linked to administrative data, did not have a prior diagnosis of diabetes, and reported not being pregnant at the time of survey. We further excluded participants who lived in a region that did not administer the depression questionnaire (n = 12,209), those who responded to the survey by proxy (n = 1648), and adults missing follow-up information due to not being eligible for health insurance at the time of survey (n = 113), no contact with the healthcare system during follow-up (n = 268), or an unclear date of death (n = 140). Finally, we excluded participants who were missing information on depression or depression-related weight change (n = 463) and adults whose BMI was not calculated as they reported a height of less than three feet or over seven feet (n = 3) (Supplementary Fig. S1 online). The primary analytic sample included 59,315 participants.

### Depression-related weight change

We first ascertained the presence of a depressive episode in the past 12 months from survey data using the Composite International Diagnostic Interview—Short Form (CIDI-SF)^20^. Participants reported whether they had experienced a 2-week period with either sadness or loss of interest in the past year^20^. Participants who endorsed either symptom were asked to report on six further symptoms during their worst depressive episode in the past 12 months. Symptom scores were summed and ranged from zero to eight symptoms. Participants with five or more symptoms were categorized as having a recent depressive episode^21^. Participants with missing information on symptoms of depression were included if their total score would be above or below the cut-off score regardless of their response to missing items but were otherwise excluded (n = 428).

Secondly, we categorized participants as experiencing depression-related weight gain, weight loss, or no weight change using the CIDI-SF question on weight change during their worst depressive episode in the past 12 months. Depression-related weight change was defined as gaining or losing at least 10 lb (4.5 kg), similar to the DSM-5 criterion of a 5% change in body weight in 1 month^7^. Participants who were missing information on symptoms of weight change or who were on a diet during their depressive episode were excluded (n = 35). As symptoms of weight change were asked only in the context of a depressive episode, we did not have information on weight change in participants who did not report a depressive episode.

### Follow-up

All participants entered the cohort on their date of survey and were followed until the first of incident type 2 diabetes, death, loss of eligibility for health insurance (e.g. by leaving the province), or the study end date of March 31, 2017. We used two administrative datasets derived from the single-payer, universal health care system in Ontario to identify these outcome measures. Firstly, the Ontario Diabetes Database (ODD) was used to identify all cases of incident diabetes using the first hospital discharge date or the first of two primary care visits with a diagnosis of diabetes, excluding gestational diabetes^22^. The ODD did not contain information on laboratory tests or medications for diabetes. Secondly, the Registered Persons’ Database (RPDB) contains all individuals with a provincial health care number and was used to identify all residents eligible for provincial health insurance for each 3-month quarter. In this study, participants who lost eligibility for health insurance (e.g., moved out of Ontario) were censored on the final day of that quarter. RPDB was also used to identify date of death. Survey and administrative datasets were linked using unique encoded identifiers and analyzed at ICES. For this work, information from administrative data was limited to the study outcomes of diabetes, death, and censoring and did not include any further information from patients’ medical records.

### Covariates

The following demographic covariates were included in the primary analysis: age group (5-year age categories as a quadratic variable), education (less than high school, high school, some post-secondary or higher), annual household income (less than $20,000, $20,000–80,000, over $80,000), marital status (married/common law or single/separated/divorced/widowed), rurality (urban or rural), birth in Canada (born inside or outside of Canada), and self-reported ethnicity (white or other). Self-reported lifestyle characteristics included smoking (current, former, or never) and alcohol consumption (frequent, occasional, former, or abstain). Physical activity levels were defined according to the Canadian Fitness and Lifestyle Institute using self-reported frequency of recreational activities lasting 15 minutes or longer and categorized as active, moderate, or inactive. Health-related covariates included self-reported hypertension and heart disease at the time of survey.

In our secondary analyses, we adjusted for attained BMI categories after a depressive episode using survey data. Attained BMI was reported at the time of survey, up to 12 months after a self-reported depressive episode. BMI was estimated using self-reported height and weight and corrected for reporting bias using Gorber’s correction equations^23^. Participants were categorized as having a BMI below 25 kg/m^2^, a BMI between 25 and 30 kg/m^2^, or a BMI of 30 kg/m^2^ and above based on their corrected BMI values^24^. We included sex-specific effects of overweight and obesity on the incidence of type 2 diabetes using an interaction term, as associations may be stronger in women^24^.

A total of 7281 (12.3%) participants were missing information on at least one covariate, with the majority missing information on household income (n = 6425). Additionally, 1216 (2.1%) participants were missing information on BMI category. All missing covariates and BMI category were imputed using fully conditional discriminant and logistic methods to create 20 complete datasets (SAS procedure mi).

### Statistical analysis

We used Cox proportional hazards regression models to estimate whether depression-related weight gain, depression-related weight loss, or depression with no weight change were associated with a higher risk of type 2 diabetes compared to no depressive episodes (SAS procedure surveyphreg). We also compared whether there were differences in diabetes incidence between adults with depression and different symptoms of weight change. The time axis was follow-up time in years after the date of survey. We calculated sex-specific associations by including an interaction term between depression-related weight change and sex. We also included an interaction term between sex and BMI categories in our secondary analysis. The proportional hazards assumption was tested by examining log–log plots and including an interaction term between depression-related weight change and follow-up time.

Results of all analyses incorporated survey weights provided by Statistics Canada, which accounted for survey design, sampling methods, and non-response^25,26^. Survey weights were normalized by dividing each participant’s survey weight by the mean of the primary sample, preserving the original sample size. Standard errors incorporated uncertainty from both survey weights and covariate imputation.

We conducted several sensitivity analyses to examine the robustness of results. We used a cut-off of three depressive symptoms to define depressive episodes and imposed no minimum requirement for weight change during a depressive episode. In order to exclude possible cases of undiagnosed diabetes at baseline, we initiated follow-up time at 1 and 5 years after participants’ survey dates. These analyses allowed us to exclude participants who were diagnosed with diabetes within 1 to 5 years after their survey date and may have had elevated glucose levels or undiagnosed diabetes at baseline. We adjusted for the use of medication for blood pressure and heart problems at baseline in participants who were asked the optional survey module on medication (n = 39,717). We further adjusted for 30-day antidepressant use at baseline, as some types of antidepressants are associated with type 2 diabetes onset^27^. Antidepressant use may also indicate the presence of other psychiatric diagnoses^28^. We compared results of unweighted proportional hazards models to Fine and Gray’s unweighted subdistribution hazard models to assess the influence of death as a competing risk. For our secondary objective, we adjusted for attained BMI as a continuous, quadratic variable in a sensitivity analysis.

## Results

The analytic sample included 59,315 participants, of whom 3965 (6.7%) reported a depressive episode in the past 12 months. Five hundred and sixty-two people (0.9%) had depression-related weight gain, while 873 (1.5%) had depression-related weight loss and 2530 (4.3%) had depression without significant weight change. Participants with depressive episodes, regardless of symptoms of weight change, were younger, more often female, had lower household income, were less likely to be married or common-law, and were more likely to identify as white (Table 1). Participants with depressive episodes were also more likely to currently smoke, had a higher prevalence of overweight and obesity, and were more likely to be using antidepressants at the time of interview compared to those without depressive episodes (Table 1). Among participants with depression, those with symptoms of weight gain were most often female, had the highest prevalence of attained overweight and obesity, and were the most likely to be using antidepressants at the time of survey (Table 1). Participants with depression-related weight loss were more often men, reported the highest prevalence of current smoking and heart disease, and had the lowest prevalence of attained overweight and obesity (Table 1).

**Table 1:** Baseline characteristics stratified by depression-related weight change in Ontario adults in the NPHS 1996, CCHS Cycle 1.1, CCHS Cycle 2.1 (n = 59,315).

	Depressive episodes with weight change			No depressive episodes
	Weight gain	No weight change	Weight loss	No depressive episodes
N	562	2530	873	55,350
**Age group % (95% CI)**				
18–24	16.8 (10.3–23.2)	18.3 (16.1–20.5)	19.2 (15.0–23.4)	12.6 (12.2–13.1)
25–34	22.0 (17.3–26.7)	22.3 (19.9–24.7)	20.4 (16.4–24.3)	18.7 (18.2–19.3)
35–44	30.5 (25.1–35.9)	27.9 (25.4–30.3)	27.6 (23.3–31.9)	24.0 (23.5–24.6)
45–54	20.4 (15.9–25.0)	19.5 (17.3–21.7)	18.0 (13.9–22.1)	18.4 (17.9–18.9)
55–64	7.6 (4.5–10.8)	7.7 (6.4–9.0)	7.3 (4.2–10.4)	11.8 (11.5–12.2)
65+	2.7 (0.3–5.0)	4.3 (3.4–5.3)	7.5 (5.0–10.0)	14.3 (14.0–14.7)
**Sex % (95% CI)**				
Female	73.2 (67.2–79.3)	68.7 (66.1–71.3)	57.5 (52.4–62.7)	50.4 (49.8–51.1)
Male	26.8 (20.7–32.8)	31.3 (28.7–33.9)	42.5 (37.3–47.6)	49.6 (48.9–50.2)
**Education level % (95% CI)**				
Less than high school	17.0 (12.8–21.3)	19.9 (17.6–22.2)	21.6 (17.2–26.0)	17.4 (17.0–17.9)
High school graduate	23.0 (17.2–28.8)	20.8 (18.6–23.0)	21.8 (17.8–25.9)	21.5 (21.0–22.0)
Post-secondary	60.0 (53.7–66.2)	59.3 (56.6–62.0)	56.6 (51.5–61.6)	61.1 (60.5–61.7)
**Annual household income % (95% CI)**				
< $20,000	16.6 (12.1–21.1)	16.8 (14.7–19.0)	19.4 (15.8–23.1)	10.9 (10.5–11.3)
$20,000–80,000	60.6 (54.3–66.9)	59.7 (56.6–62.8)	57.2 (51.8–62.6)	60.4 (59.8–61.1)
> $80,000	22.8 (17.3–28.3)	23.4 (20.7–26.2)	23.4 (18.4–28.4)	28.7 (28.1–29.3)
**Marital status % (95% CI)**				
Married or common-law	52.5 (46.1–58.8)	50.2 (47.5–53.0)	42.6 (37.5–47.7)	65.1 (64.5–65.7)
Single, separated, divorced, or widowed	47.5 (41.2–53.9)	49.8 (47.0–52.5)	57.4 (52.3–62.5)	34.9 (34.3–35.5)
**Geography % (95% CI)**				
Urban	85.2 (81.5–88.9)	90.4 (89.0–91.7)	87.2 (84.3–90.1)	86.8 (86.4–87.1)
Rural	14.8 (11.1–18.5)	9.6 (8.3–11.0)	12.8 (9.9–15.7)	13.2 (12.9–13.6)
**Ethnicity % (95% CI)**				
White	90.9 (87.1–94.7)	86.4 (84.1–88.7)	85.9 (81.4–90.5)	83.4 (82.8–83.9)
Other	9.1 (5.3–12.9)	13.6 (11.3–15.9)	14.1 (9.5–18.6)	16.6 (16.1–17.2)
**Born in Canada % (95% CI)**				
Born in Canada	79.0 (73.4–84.6)	75.3 (72.6–78.0)	77.9 (73.1–82.7)	69.3 (68.6–69.9)
Born outside of Canada	21.0 (15.4–26.6)	24.7 (22.0–27.4)	22.1 (17.3–26.9)	30.7 (30.1–31.4)
**Smoking status % (95% CI)**				
Current	39.6 (33.3–45.9)	40.5 (37.8–43.1)	47.7 (42.7–52.8)	25.3 (24.7–25.8)
Former	35.6 (29.6–41.6)	31.4 (28.9–33.9)	30.1 (25.1–35.1)	35.7 (35.1–36.3)
Never	24.8 (19.7–30.0)	28.1 (25.5–30.7)	22.2 (17.6–26.8)	39.0 (38.4–39.6)
**Alcohol consumption % (95% CI)**				
Frequent	56.4 (50.3–62.5)	59.9 (57.2–62.6)	57.7 (52.6–62.8)	62.8 (62.2–63.4)
Occasional	23.1 (18.1–28.1)	23.5 (21.1–25.8)	20.2 (16.4–24.0)	18.4 (17.9–18.9)
Former	15.8 (11.6–20.0)	10.8 (9.3–12.4)	15.9 (12.1–19.7)	10.7 (10.4–11.1)
Abstain	4.7 (2.4–6.9)	5.8 (4.1–7.5)	6.2 (2.7–9.7)	8.1 (7.7–8.5)
**Physical activity level % (95% CI)**				
Active	17.6 (13.1–22.1)	21.4 (19.1–23.7)	23.2 (18.9–27.4)	21.5 (21.0–22.0)
Moderate	26.0 (20.1–31.9)	22.4 (20.2–24.6)	21.2 (17.2–25.2)	24.0 (23.5–24.5)
Inactive	56.4 (50.1–62.7)	56.2 (53.5–58.9)	55.6 (50.6–60.7)	54.5 (53.9–55.2)
**High blood pressure % (95% CI)**				
Yes	13.6 (8.6–18.7)	9.4 (7.9–10.9)	10.9 (7.6–14.2)	12.3 (12.0–12.7)
No	86.4 (81.3–91.4)	90.6 (89.1–92.1)	89.1 (85.8–92.4)	87.7 (87.3–88.0)
**Heart disease % (95% CI)**				
Yes	2.5 (1.3–3.7)	4.8 (3.8–5.9)	6.5 (2.9–10.2)	4.3 (4.1–4.5)
No	97.5 (96.3–98.7)	95.2 (94.1–96.2)	93.5 (89.8–97.1)	95.7 (95.5–95.9)
**BMI category % (95% CI)**^a^				
Under 25 kg/m^2^	26.2 (20.1–32.2)	47.8 (45.0–50.6)	57.9 (52.9–62.9)	42.4 (41.8–43.0)
25–30 kg/m^2^	36.4 (30.3–42.6)	31.9 (29.3–34.6)	29.0 (24.5–33.4)	38.5 (37.9–39.1)
30 kg/m^2^ and above	37.4 (31.5–43.4)	20.3 (18.1–22.4)	13.2 (9.7–16.6)	19.2 (18.7–19.6)
**Antidepressant use % (95% CI)**^b^				
Yes	43.7 (36.2–51.1)	23.8 (21.0–26.6)	29.2 (24.0–34.4)	2.9 (2.7–3.1)
No	56.3 (48.9–63.8)	76.2 (73.4–79.0)	70.8 (65.6–76.0)	97.1 (96.9–97.3)
**Medication for blood pressure % (95% CI)**^b^				
Yes	6.6 (3.6–9.6)	6.6 (4.9–8.2)	8.5 (4.8–12.2)	10.3 (9.9–10.7)
No	93.4 (90.4–96.4)	93.4 (91.8–95.1)	91.5 (87.8–95.2)	89.7 (89.3–90.1)
**Medication for heart problems % (95% CI)**^b^				
Yes	3.8 (1.2–6.3)	3.7 (2.5–4.9)	5.4 (1.4–9.5)	4.4 (4.2–4.7)
No	96.2 (93.7–98.8)	96.3 (95.1–97.5)	94.6 (90.5–98.6)	95.6 (95.3–95.8)

Weighted using survey weights provided by Statistics Canada; all missing covariates imputed.

^**a**^BMI categories corrected using Gorber’s correction equations.

^b^N = 39,717, excluding participants not asked optional medication module (n = 19,552) or missing information on medication (n = 45).

**Figure 1:**
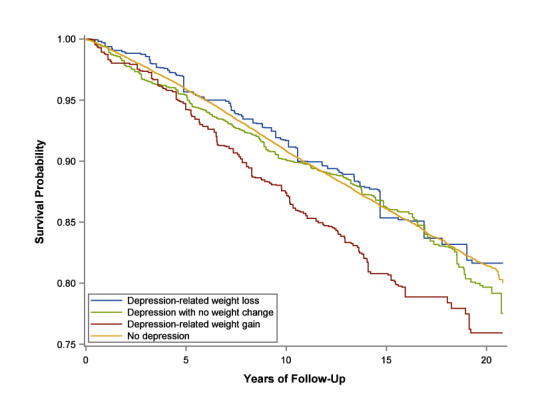
Weighted Kaplan–Meier survival estimates for risk of type 2 diabetes stratified by depression-related weight change for Ontario adults in the NPHS 1996, CCHS Cycle 1.1, CCHS Cycle 2.1 (n = 59,315).

Follow-up time ranged from less than 1 year to over 20 years, with a median of 15.6 years and an interquartile range of 13.4 to 16.5 years. A total of 8543 participants (14.4%) were diagnosed with diabetes during follow-up. Diabetes incidence rates were highest in participants with depression-related weight gain and were similar among other participants (Fig. 1; Table 2). When adjusting for all covariates, depression-related weight gain was associated with an increased risk of diabetes compared to no depressive episodes (HR 1.70, 95% CI 1.32–2.20) (Table 3). Depression with no weight change was also associated with a 23% increased risk of type 2 diabetes compared to no depression (HR 1.23, 95% CI 1.04–1.45). However, depression with weight loss was not associated with an increased risk of type 2 diabetes. When comparing adults with depression, depression-related weight gain was associated with a higher incidence of type 2 diabetes compared to depression-related weight loss (HR 1.62, 95% CI 1.09–2.42) and depression with no weight change (HR 1.39, 95% CI 1.03–1.86) (Table 3). We did not observe meaningful differences between depression with no weight change and depression-related weight loss (HR depression with no weight change vs. weight loss 1.17, 95% 0.83–1.66). The proportional hazards assumption was met when examining log–log plots and including interaction terms between depression-related weight change and follow-up time (Supplementary Fig. S2 online).

**Table 2:** Incidence of death, type 2 diabetes, and follow-up time stratified by depression-related weight change for Ontario adults in the NPHS 1996, CCHS Cycle 1.1, CCHS Cycle 2.1 (n = 59,315).

Depressive episodes	N	Cumulative incidence of diabetes (95% CI)	Incidence rates of diabetes per 1000 person-years (95% CI)	Median follow-up time (interquartile range)
Depression-related weight gain	562	19.50 (15.33–23.67)	14.08 (11.62–17.07)	15.55 (13.37–16.20)
Depression with no weight change	2530	14.24 (12.18–16.30)	10.09 (9.08–11.21)	15.63 (13.43–16.30)
Depression-related weight loss	873	13.40 (9.09–17.71)	9.67 (7.95–11.76)	15.53 (13.32–16.28)
No depression	55,350	14.28 (13.85–14.71)	9.98 (9.77–10.21)	15.61 (13.35–16.48)

Weighted using survey weights provided by Statistics Canada.

**Table 3:** Multivariable adjusted hazard models for depression-related weight change and incident type 2 diabetes for Ontario adults in the NPHS 1996, CCHS Cycle 1.1, CCHS Cycle 2.1 (n = 59,315).

Depressive episodes	Total population		Men		Women	
	HR adjusted for age and sex (95% CI)	HR fully-adjusted^a^ (95% CI)	HR adjusted for age (95% CI)	HR fully-adjusted^a^ (95% CI)	HR adjusted for age (95% CI)	HR fully-adjusted^a^ (95% CI)
Depression-related weight gain versus no depression	1.79 (1.39–2.31)	1.70 (1.32–2.20)	1.57 (0.93–2.65)	1.46 (0.86–2.47)	1.89 (1.43–2.51)	1.82 (1.38–2.41)
Depression with no weight change versus no depression	1.30 (1.10–1.52)	1.23 (1.04–1.45)	1.29 (0.99–1.68)	1.20 (0.92–1.56)	1.30 (1.06–1.59)	1.24 (1.01–1.53)
Depression-related weight loss versus no depression	1.20 (0.87–1.65)	1.05 (0.77–1.43)	1.55 (1.01–2.37)	1.34 (0.87–2.09)	0.91 (0.56–1.49)	0.81 (0.52–1.26)
Depression-related weight gain versus weight loss	1.50 (1.00–2.24)	1.62 (1.09–2.42)	1.02 (0.52–1.99)	1.09 (0.55–2.15)	2.07 (1.18–3.63)	2.26 (1.34–3.81)
Depression-related weight gain versus no weight change	1.38 (1.03–1.86)	1.39 (1.03–1.86)	1.22 (0.68–2.18)	1.22 (0.68–2.18)	1.46 (1.04–2.05)	1.47 (1.04–2.06)
Depression with no weight change versus weight loss	1.08 (0.76–1.54)	1.17 (0.83–1.66)	0.83 (0.51–1.37)	0.89 (0.54–1.48)	1.42 (0.84–2.40)	1.54 (0.95–2.51)

Weighted using survey weights provided by Statistics Canada; all missing covariates imputed.

^a^Estimates adjusted for age categories, sex, education, income, marital status, geography, born in Canada, ethnicity, smoking, alcohol consumption, physical activity levels, self-reported hypertension and heart disease, and survey cycle.

Sex-stratified results showed that among women, depression-related weight gain was associated with an increased risk of type 2 diabetes compared to no depression (HR 1.82, 95% CI 1.38–2.41) (Table 3). Depression with no weight change was also associated with a 24% increased risk of type 2 diabetes compared to no depression (HR 1.24, 95% CI 1.01–1.53). When comparing women with depression, depression-related weight gain was associated with an increased risk of diabetes compared to depression-related weight loss (HR 2.26, 95% CI 1.34–3.81) and depression with no weight change (HR 1.47, 95% 1.04–2.06). Among men, smaller associations were observed between depression and type 2 diabetes and the variance in all estimates was high. Interaction terms between sex and depression-related weight gain were wide (HR interaction 1.66 for weight gain in women vs. men, 95% CI 0.89–3.11), indicating some uncertainty in sex differences.

In our secondary analysis, the increased risk of type 2 diabetes in adults with depression-related weight gain compared to no depression was smaller when adjusting for attained overweight and obesity (HR 1.38, 95% CI 1.08–1.78) (Table 4). Among women, depression-related weight gain was associated with a 51% increased risk of type 2 diabetes (HR 1.51, 95% CI 1.15–1.98) compared to no depression. Among men, the point estimate suggested that depression-related weight loss was associated with a 54% increased risk of type 2 diabetes (HR 1.54, 95% CI 0.99–2.37), though the variance in the estimate was high. Results of sensitivity analyses were consistent with the main results (Supplementary Tables S1–S3 online).

**Table 4:** Multivariable adjusted hazard models for depression-related weight change and incident type 2 diabetes adjusting for attained BMI category for Ontario adults in the NPHS 1996, CCHS Cycle 1.1, CCHS Cycle 2.1 (n = 59,315).

Depressive episodes	Total	Men	Women
	HR fully-adjusted^a^ (95% CI)	HR fully-adjusted^a^ (95% CI)	HR fully-adjusted^a^ (95% CI)
Depression-related weight gain versus no depression	1.38 (1.08–1.78)	1.15 (0.68–1.95)	1.51 (1.15–1.98)
Depression with no weight change versus no depression	1.22 (1.04–1.43)	1.19 (0.92–1.54)	1.23 (0.99–1.51)
Depression-related weight loss versus no depression	1.26 (0.91–1.74)	1.54 (0.99–2.37)	1.01 (0.62–1.65)
Depression-related weight gain versus weight loss	1.10 (0.73–1.64)	0.75 (0.38–1.48)	1.48 (0.86–2.57)
Depression-related weight gain versus no weight change	1.14 (0.85–1.53)	0.97 (0.54–1.73)	1.22 (0.88–1.71)
Depression with no weight change versus weight loss	0.96 (0.68–1.37)	0.78 (0.47–1.28)	1.21 (0.72–2.04)

Weighted using survey weights provided by Statistics Canada; all missing covariates imputed.

^a^Estimates adjusted for age categories, sex, education, income, marital status, geography, born in Canada, ethnicity, smoking, alcohol consumption, physical activity levels, self-reported hypertension and heart disease, survey cycle, and attained BMI category.

## Discussion

These findings provide evidence that the risk of type 2 diabetes differs in adults with depressive episodes with symptoms of weight gain, weight loss, or no weight change compared to adults with no depression. In a population-based sample, adults with depression-related weight gain had a 70% increased risk of type 2 diabetes over 20 years compared to those with no depression. Adults with depression but no symptoms of significant weight change had a 23% increased risk. Conversely, adults with depression with symptoms of weight loss were not at increased risk of type 2 diabetes. These results also suggest sex-specific associations, as depression-related weight gain was associated with incident type 2 diabetes among women only. Assessing symptoms of weight change in depression may aid in identifying adults with depression at highest risk of type 2 diabetes, particularly among women.

The results of this study are consistent with previous evidence that suggests that individual symptoms of depression may be used to identify adults at higher risk of metabolic health outcomes. In a sample of adults with major depressive disorder, those with increased appetite had higher insulin resistance compared to adults with no depression and compared to adults with depression and appetite loss^6^. In the Netherlands Study of Depression and Anxiety, adults with depression that included increased appetite had higher BMI, higher waist circumference, and more metabolic syndrome criteria^5^. Our findings suggest that it may be important to consider symptoms of weight change in addition to appetite change, as not all adults with appetite change also experience weight change. In a sample of psychiatric outpatients, adults with symptoms of increased appetite had a 70% probability of also experiencing symptoms of significant weight gain^29^. Overall, our findings extend prior research on specific depressive symptoms by showing that symptoms of weight change may identify adults with depression at higher risk of type 2 diabetes.

These results are also consistent with longitudinal evidence that specific depressive subtypes, notably those including symptoms of weight gain or increased appetite, are associated with poorer metabolic outcomes^8,10,30^. Lasserre et al. reported that adults with lifetime depressive disorders with atypical features had steeper increases in fasting glucose over 5 years when compared to adults with no history of depression^8^. Adults with depressive disorders with melancholic features, including reduced appetite, or depressive disorders that were neither atypical nor melancholic, showed smaller and more variable increases in fasting glucose^8^. Furthermore, two studies have reported that atypical depression is associated with an increased incidence of metabolic syndrome compared to no depression^8,10^. Our results show longitudinal associations between depression-related weight gain, depression without significant weight change, and the incidence of type 2 diabetes over up to 20 years. Furthermore, results from our secondary objective found that depression-related weight gain and depression with no weight change were associated with type 2 diabetes incidence even when accounting for attained overweight and obesity.

There are several potential biological mechanisms between depression with weight gain and the development of type 2 diabetes. Some evidence, though not all^31^, has reported that atypical depression is associated with markers of immunometabolic dysregulation such as increased C-reactive protein, TNFα and leptin^30^. Weight gain or increased appetite in depression are also associated with biomarkers of poor immune functioning and inflammation^5,6,30^. Our results are consistent with a proposed immunometabolic subtype of depression characterized by symptoms of increased appetite, weight gain, fatigue, hypersomnia, and leaden paralysis^30,32^. This subtype of depression is hypothesized to be linked to cardiometabolic conditions such as obesity, metabolic syndrome, and diabetes^30,32^.

Our results are also the first to present associations between depression-related weight change and incident type 2 diabetes stratified by sex. Depression-related weight gain was associated with an increased risk of type 2 diabetes among women only. Other work examining depressive subtypes or individual depressive symptoms and metabolic parameters did not stratify by sex^5,8,10^ or did not find cross-sectional evidence of sex differences in statistical models^31^. As depressive symptoms of weight gain and increased appetite appear more common in women^4,11,33^, identifying depression-related weight gain may be especially important for risk assessment and diabetes prevention among women.

It should be noted that this study does not provide evidence that weight gain in depression is more strongly associated with type 2 diabetes incidence than weight gain in the general population. Our study design did not allow us to compare depression-related weight change with weight change for other reasons. There is evidence that long-term weight change during adulthood is associated with diabetes incidence even when accounting for attained BMI^16,17^. Consistent with these findings, our study demonstrated that weight gain in the context of depression was also associated with type 2 diabetes incidence when adjusting for attained BMI. Central adiposity may contribute to this association, as prior evidence reports that depression is associated with increased central obesity and higher waist circumference among adults with comorbid diabetes^34,35^. Measures of central adiposity have also been associated with diabetes incidence independently of BMI^36,37^.However, further information is needed comparing depression-related weight gain to weight gain that is not associated with depression to better understand the impacts of depression and weight gain on metabolic outcomes.

There are several strengths to this study. It is the first to compare depression-related weight change and incident type 2 diabetes in a large, community-based sample. Our median follow-up time of over 15 years allowed sufficient opportunity to observe the development and diagnosis of diabetes, which may take several years^38^. Our population-based sample of community-dwelling adults overcomes limitations from previous studies that used smaller or highly-selected clinical samples^5,6,39^. We conducted several sensitivity analyses to examine whether our results were influenced by the definition of depression, undiagnosed diabetes, or potential confounders.

Several limitations should be considered when interpreting these results. Due to limitations of the CIDI-SF, we could not estimate associations between other symptoms of depression and incidence of type 2 diabetes, notably symptoms of increased or decreased appetite. Our outcome measure could not distinguish between incident type 1 and type 2 diabetes, though the vast majority of diabetes cases diagnosed after age 30 are type 2 diabetes^40^. The outcome was limited to physician-diagnosed diabetes only and did not include laboratory measures of glucose levels or HbA1c or use of medication for diabetes. The prevalence of undiagnosed diabetes was estimated at 3.4% among the Canadian population from 2009 to 2011^41^. However, our considerable follow-up time likely minimized the impact of undiagnosed diabetes and diagnostic delays in this study. There may also be unmeasured confounding as we could not adjust for other psychiatric comorbidities, medication use, or clinical measures such as blood pressure values and cholesterol levels. Nonetheless, we have adjusted for all confounders associated with diabetes in a risk prediction model in Canada^42^. There may be misclassification of hypertension and heart disease due to underreported or undiagnosed conditions, although agreement between self-report and administrative data for these conditions has been reported as moderate to good^43,44^. Finally, we only had information on weight change for study participants who reported a recent depressive episode. We therefore could not compare diabetes incidence between adults with depression-related weight change and weight change unrelated to depression.

These results show that adults with depression-related weight gain or depression with no weight change had a higher risk of type 2 diabetes over up to 20 years compared to adults without depression. Adults with depression-related weight loss did not have a higher risk of type 2 diabetes. Associations were more prominent among women compared to men and persisted after accounting for attained overweight and obesity. Overall, these findings indicate that weight change, as a symptom of depression, may be an indication of long-term risk for type 2 diabetes, particularly among women. These findings can be applied to aid in identifying adults with depression who are at highest risk of type 2 diabetes. They can also contribute to developing tailored diabetes prevention strategies for adults with depression. These findings further support a heterogeneous concept of depression with a potential immunometabolic subtype, characterised by symptoms of increased appetite and weight gain as well as metabolic dysregulation^5,30,32^.

## Supplementary Information

Supplementary Information.

## Data Availability

The dataset from this study is held securely in coded form at ICES. While legal data sharing agreements between ICES and data providers (e.g., healthcare organizations and government) prohibit ICES from making the dataset publicly available, access may be granted to those who meet pre-specified criteria for confidential access, available at www.ices.on.ca/DAS (email: das@ices.on.ca).
