# Supplementary material for: Depression-related weight change and incident diabetes in a community sample
Source: Sci Rep. 2021 Jun 30;11:13575. doi: 10.1038/s41598-021-92963-w (PMC8245524; doi:10.1038/s41598-021-92963-w)
Supplement: Supplementary file 1 — Supplementary Information. [file 41598_2021_92963_MOESM1_ESM.docx]

**Supplementary Information**

**Title of Manuscript: Depression-Related Weight Change and Incident Diabetes in a Community Sample**

Authors: E. Graham, T. Watson, S. S. Deschênes, K. B. Filion, M. Henderson, S. Harper, L. C. Rosella, N. Schmitz

**Supplementary Figure S1:** Participant Flow Diagram


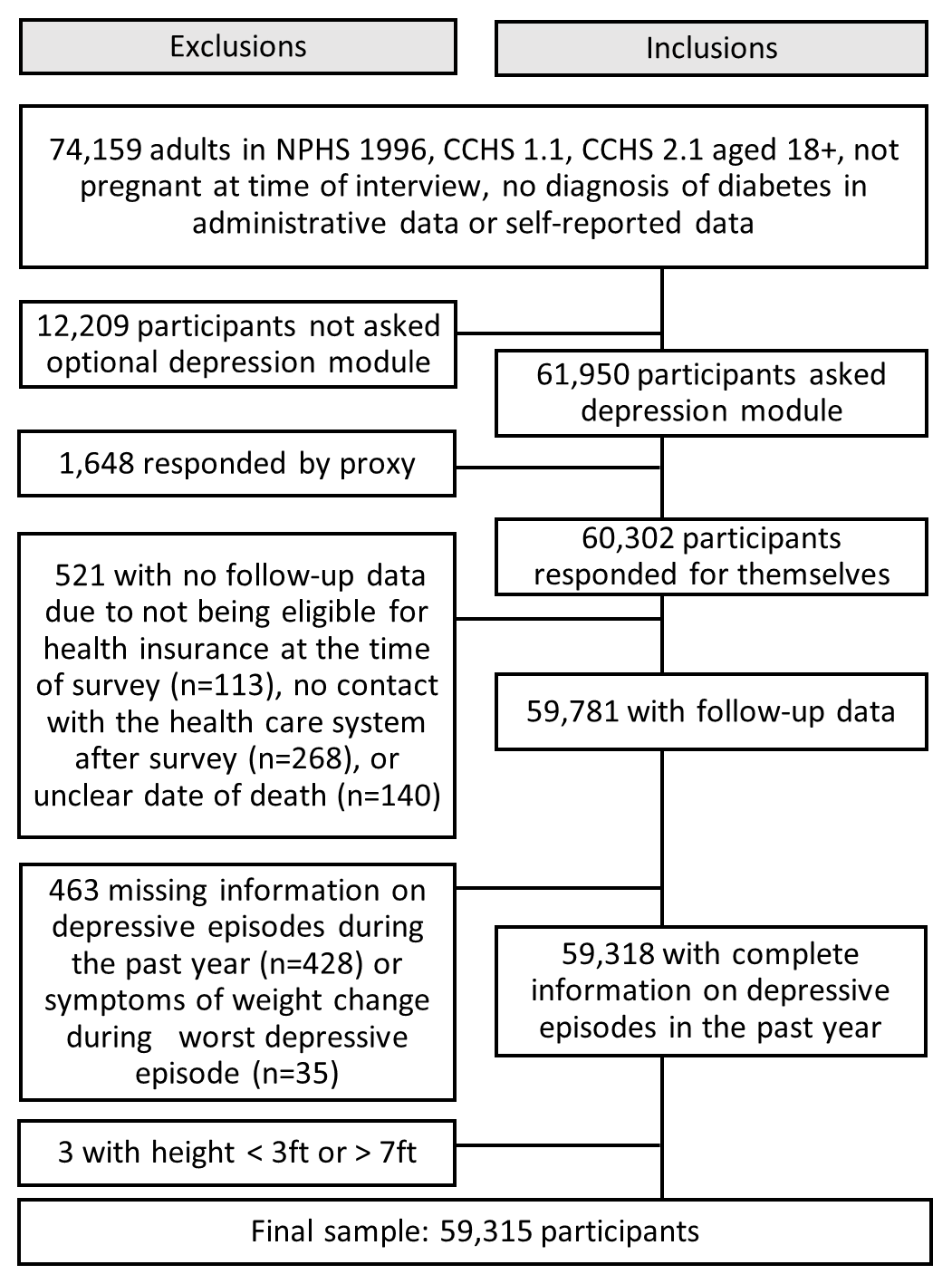


**Supplementary Figure S2:** Weighted Log-Log Plots for Hazard of Diabetes Stratified by Depression-Related Weight Change for Ontario Adults in the NPHS 1996, CCHS Cycle 1.1, CCHS Cycle 2.1 (n=59,315)


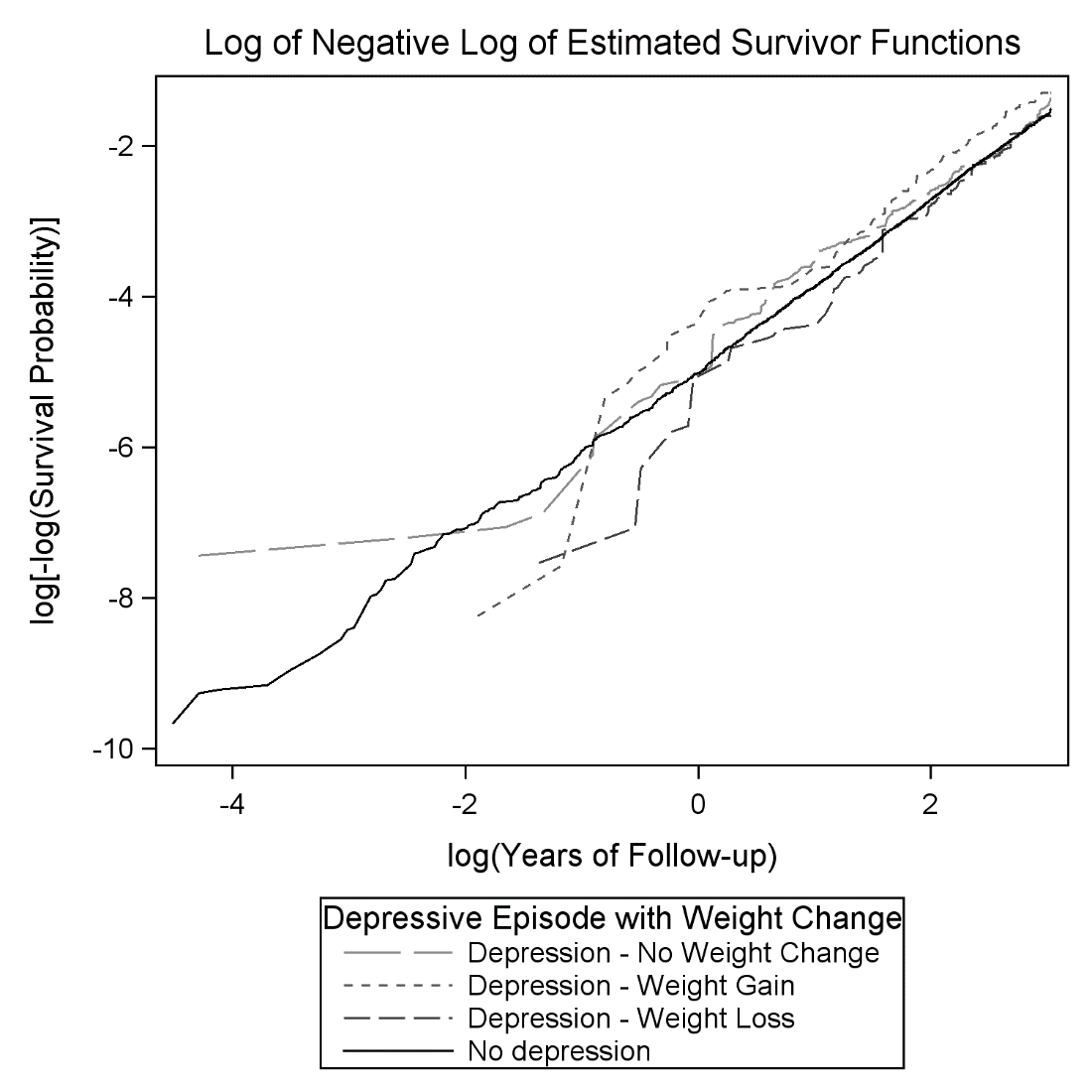


**Supplementary Table S1:** Sensitivity Analyses of Exposure Definitions and Covariates for Multivariable Adjusted Hazard Models of Depression-Related Weight Change and Incident Type 2 Diabetes for Ontario Adults in the NPHS 1996, CCHS Cycle 1.1, CCHS Cycle 2.1*

| **Depressive episodes** | **CIDI-SF cut-off of 3**† | **No minimum weight change**† | **Adjusted for medication for hypertension and heart problems** †**‡** | **Adjusted for medication for hypertension, heart problems, and antidepressants**†**‡** |
| --- | --- | --- | --- | --- |
| **N** | 59,310 | 59,315 | 39,717 | 39,717 |
| Depression-related weight gain versus no depression | 1.70 (1.33-2.18) | 1.55 (1.24-1.92) | 1.73 (1.25-2.41) | 1.58 (1.13-2.21) |
| Depression with no weight change versus no depression | 1.18 (1.02-1.35) | 1.24 (1.03-1.48) | 1.18 (0.98-1.43) | 1.12 (0.92-1.37) |
| Depression-related weight loss versus no depression | 1.02 (0.75-1.37) | 1.10 (0.85-1.42) | 1.15 (0.81-1.63) | 1.08 (0.75-1.56) |
| Depression-related weight gain versus weight loss | 1.67 (1.14-2.46) | 1.41 (1.01-1.97) | 1.51 (0.94-2.43) | 1.46 (0.90-2.36) |
| Depression-related weight gain versus no weight change | 1.45 (1.10-1.91) | 1.25 (0.95-1.65) | 1.46 (1.01-2.13) | 1.41 (0.96-2.05) |
| Depression with no weight change versus weight loss | 1.16 (0.84-1.61) | 1.13 (0.83-1.54) | 1.03 (0.69-1.53) | 1.04 (0.70-1.55) |

*Weighted using survey weights provided by Statistics Canada; all missing covariates imputed

**†**Estimates adjusted for age categories, sex, education, income, marital status, geography, born in Canada, ethnicity, smoking, alcohol consumption, physical activity levels, self-reported hypertension and heart disease, and survey cycle

‡n=39,717, excluding participants not asked optional medication module (n=19,552) or missing information on medication (n=45)

**Supplementary Table S2:** Sensitivity Analyses of Outcomes for Multivariable Adjusted Hazard Models of Depression-Related Weight Change and Incident Type 2 Diabetes for Ontario Adults in the NPHS 1996, CCHS Cycle 1.1, CCHS Cycle 2.1*

| **Depressive episodes** | **1-year lag period for follow-up**† | **5-year lag period for follow-up**† | **Unweighted model**† | **Unweighted competing risks model**† |
| --- | --- | --- | --- | --- |
| N | 58,415 | 53,586 | 59,315 | 59,315 |
| Depression-related weight gain versus no depression | 1.66 (1.28-2.16) | 1.67 (1.25-2.23) | 1.70 (1.32-2.20) | 1.88 (1.57-2.24) |
| Depression with no weight change versus no depression | 1.23 (1.04-1.45) | 1.16 (0.95-1.40) | 1.23 (1.04-1.45) | 1.10 (0.99-1.23) |
| Depression-related weight loss versus no depression | 1.05 (0.76-1.45) | 1.01 (0.69-1.48) | 1.05 (0.77-1.43) | 0.82 (0.67-1.01) |
| Depression-related weight gain versus weight loss | 1.59 (1.05-2.40) | 1.65 (1.03-2.65) | 1.62 (1.09-2.42) | 2.28 (1.75-2.97) |
| Depression-related weight gain versus no weight change | 1.35 (1.00-1.84) | 1.44 (1.03-2.03) | 1.39 (1.03-1.86) | 1.70 (1.39-2.09) |
| Depression with no weight change versus weight loss | 1.17 (0.82-1.68) | 1.15 (0.75-1.74) | 1.17 (0.83-1.66) | 1.34 (1.06-1.68) |

*Weighted using survey weights provided by Statistics Canada; all missing covariates imputed

**†**Estimates adjusted for age categories, sex, education, income, marital status, geography, born in Canada, ethnicity, smoking, alcohol consumption, physical activity levels, self-reported hypertension and heart disease, and survey cycle

**Supplementary Table S3:** Sensitivity Analysis Adjusting for Continuous BMI Values for Multivariable Adjusted Hazard Models of Depression-Related Weight Change and Incident Type 2 Diabetes for Ontario Adults in the NPHS 1996, CCHS Cycle 1.1, CCHS Cycle 2.1 (n=59,315)*

| **Depressive episodes** | **Total** | **Men** | **Women** |
| --- | --- | --- | --- |
|  | **Adjusted for attained BMI value†** | **Adjusted for attained BMI value†** | **Adjusted for attained BMI value†** |
| Depression-related weight gain versus no depression | 1.21 (0.93-1.57) | 1.02 (0.59-1.75) | 1.31 (0.99-1.74) |
| Depression with no weight change versus no depression | 1.20 (1.02-1.41) | 1.15 (0.89-1.50) | 1.22 (1.00-1.50) |
| Depression-related weight loss versus no depression | 1.30 (0.95-1.77) | 1.59 (1.02-2.47) | 1.04 (0.67-1.61) |
| Depression-related weight gain versus weight loss | 0.93 (0.62-1.39) | 0.64 (0.32-1.28) | 1.26 (0.75-2.11) |
| Depression-related weight gain versus no weight change | 1.01 (0.75-1.37) | 0.88 (0.48-1.60) | 1.07 (0.77-1.50) |
| Depression with no weight change versus weight loss | 0.92 (0.65-1.30) | 0.73 (0.44-1.21) | 1.17 (0.73-1.89) |

*Weighted using survey weights provided by Statistics Canada; all missing covariates imputed

**†**Estimates adjusted for age categories, sex, education, income, marital status, geography, born in Canada, ethnicity, smoking, alcohol consumption, physical activity levels, self-reported hypertension and heart disease, survey cycle,

and quadratic BMI value
